# Identification of Clinical Factors Associated With the Immunogenicity of Homologous ChAdOx1‐nCoV‐19 Vaccine in Hemodialysis Patients

**DOI:** 10.1002/kjm2.70173

**Published:** 2026-01-19

**Authors:** Chia‐Wei Chang, Ping‐Hsun Wu, Li‐Yun Chang, Yu‐Ying Huang, Teng‐Hui Huang, Su‐Chu Lee, Yi‐wen Chiu, Shang‐Jyh Hwang, Tsun‐Yung Kuo, Mei‐Chuan Kuo

**Affiliations:** ^1^ Department of Medical Education Kaohsiung Chang Gung Memorial Hospital Kaohsiung Taiwan; ^2^ Division of Nephrology, Department of Internal Medicine Kaohsiung Medical University Hospital, Kaohsiung Medical University Kaohsiung Taiwan; ^3^ Faculty of Medicine, College of Medicines Kaohsiung Medical University Kaohsiung Taiwan; ^4^ Center for Big Data Research Kaohsiung Medical University Kaohsiung Taiwan; ^5^ Biomedical Artificial Intelligence Academy Kaohsiung Medical University Kaohsiung Taiwan; ^6^ Department of Nursing Kaohsiung Medical University Hospital, Kaohsiung Medical University Kaohsiung Taiwan; ^7^ Department of Biotechnology and Animal Science National Ilan University Yilan City Yilan County Taiwan

**Keywords:** antibody responses, COVID‐19, hemodialysis, immune responses, vaccine

## Abstract

Hemodialysis (HD) patients are at higher risk of severe COVID‐19 and may exhibit suboptimal vaccine responses. This study evaluates the factors influencing vaccine‐induced immunity in HD patients following the second dose of the Vaxzevria. A total of 276 HD patients and 126 controls were included. Antibody responses were assessed using binding antibody units (BAU). Operational antibody thresholds used in prior literature were applied to classify antibody levels as evaluated as 50% and 60% vaccine efficacy (VE) levels after the first dose and second dose separately. Linear regression model and stratification by baseline characteristics were performed to identify clinical factors associated with vaccine responses. HD patients had significantly lower median antibody levels compared to healthy subjects. Antibody levels decreased with age, with the oldest tertile having the lowest response. Higher BMI and favorable body composition, such as increased fat tissue index, were positively correlated with stronger immune responses. Patients with elderly, diabetes mellitus, heart failure, and low BMI were associated with reduced antibody responses and lower rates of achieving 50% and 60% VE thresholds. Vaccine‐induced immunity in HD patients is influenced by age, BMI, body composition, and comorbidities. Tailored vaccination strategies, including booster doses, are essential to enhance protection in this vulnerable population.

## 1 Introduction

The range of COVID‐19 manifestations spans from asymptomatic cases to severe multiorgan failure, characterized by hyperinflammation and a hypercoagulable state, potentially leading to fatal outcomes [1, 2]. Patients diagnosed with end‐stage kidney disease (ESKD) are classified as immunocompromised individuals and exhibit increased susceptibility to elevated morbidity or mortality when infected with SARS‐CoV‐2 during the COVID‐19 pandemic [3, 4, 5]. Vaccination has been shown to effectively reduce the severity and mortality of COVID‐19 infection among ESKD patients [6, 7]. However, a less effective antibody response was observed in ESKD patients when compared to those in healthy individuals [8, 9, 10]. Furthermore, a limited antibody response to the viral vector COVID‐19 vaccine was found in hemodialysis patients [11]. In another systemic review, evidence is presented indicating reduced seroconversion and seroprotection rates post‐vaccination for viral respiratory diseases in hemodialysis patients [12]. In addition, dialysis patients presented a significant decrease in antibodies after successful SARS‐CoV‐2 mRNA vaccination compared to non‐dialysis chronic kidney disease patients [13].

Vaccination responses can differ among individuals and are usually poor responses in immunocompromised subjects. According to the previous study, many factors may lower the immune response to vaccines, such as the elderly, the use of immunosuppressive therapy or chemotherapy, or dialysis treatment [14, 15]. Other biochemical factors, including lower serum albumin levels, decreased white blood cell or lymphocyte counts, and lower hemoglobin levels, were also recognized as factors associated with a diminished antibody response or lack of response [10, 14].

It is widely recognized that dialysis patients or kidney transplant subjects exhibit a diminished immune response to various vaccines, including those for hepatitis B and the influenza A virus subtype H1N1. However, to date, studies examining contributing factors to poor responses of SARS‐CoV‐2 vaccination in hemodialysis patients remain limited, so we aim to determine the immune response following COVID‐19 vaccination with Vaxzevria at the first dose and second dose and examine the clinical factors that could influence the humoral response.

Although vaccine immunogenicity in hemodialysis (HD) patients has been examined in several Western cohorts, data from Asian HD populations receiving the ChAdOx1 nCoV‐19 vaccine remain limited. Moreover, prior studies have typically evaluated demographic or biochemical predictors in isolation. Few investigations have simultaneously analyzed BMI, detailed body composition parameters such as fat tissue index (FTI), total body water (TBW), and extracellular‐to‐intracellular water ratio (ECW/ICW), together with serologic vaccine response within the same HD cohort. This integrated approach is important because nutritional status, hydration status, and inflammation are highly interrelated in chronic dialysis patients and may jointly influence immunogenicity.

In this context, our study contributes novel evidence by evaluating these interdependent factors using a large and representative Taiwanese HD cohort (*n* = 276), providing population‐specific insights that have been underreported in the literature.

## 2 Methods

### 2.1 Study Participants

This study was approved by the Institutional Review Board of Kaohsiung Medical University (KMUHIRB‐E(II)‐20210161). Informed consent was obtained from all participants. Hemodialysis (HD) patients aged over 18 years with ≥ 90 days of maintenance HD were recruited from May to October 2021. All received thrice‐weekly high‐efficiency HD (Kt/V > 1.2). Non‐dialysis healthy controls were also enrolled, excluding those with kidney disease. Demographic and clinical data were obtained from electronic records and self‐report. Baseline comorbidities, BMI, and biochemical profiles within 30 days before the second vaccine dose were collected. BMI was grouped into low (< 24), middle [16, 17, 18, 19], and high (≥ 27 kg/m^2^).

Body composition was assessed using bioimpedance spectroscopy (Body Composition Monitor, BCM; Fresenius Medical Care). A total of 276 hemodialysis patients underwent BCM measurement, representing a convenience subsample based on equipment availability and scheduling feasibility at the dialysis unit. Because BCM assessment was not routinely performed for all patients during the study period, body‐composition data were not available for the entire cohort.

We acknowledge that the reliance on a subsample may introduce selection bias, particularly if patients who underwent BCM assessment differed from those who did not. Baseline characteristics of the non‐BCM group could not be compared due to limited available data, and therefore the representativeness of the BCM subsample cannot be fully established. Consequently, the findings related to FTI, TBW, and ECW/ICW should be interpreted cautiously and considered exploratory.

This study included all eligible hemodialysis patients receiving ChAdOx1 vaccination during the predefined enrollment period; therefore, no a priori sample size calculation was performed. As a post hoc assessment, the final sample size provided > 80% power to detect the observed effect sizes for age, diabetes, and BMI on antibody response at a two‐sided alpha of 0.05.

### 2.2 Antibody Titer Measurement

Serum IgG titers against SARS‐CoV‐2 spike protein were measured at 8 weeks after the first and 2 weeks after the second dose of ChAdOx1 nCoV‐19 vaccine using a commercial ELISA kit. Samples were processed and analyzed per manufacturer instructions, with titers converted to BAU/mL using WHO standards. For BAU/mL conversion, the WHO NIBSC 20/136 reference standard was tested by the same ELISA assay, and the anti‐spike IgG titer for NIBSC 20/136 was 10,960.9. Since the NIBSC 20/136 reference standard corresponds to 1000 BAU/mL, a conversion factor of 0.0912 (1000/10,960.9) was applied to estimate BAU/mL values in this study [20].

To contextualize antibody levels relative to expected clinical protection, we applied commonly referenced operational thresholds derived from prior studies evaluating correlates of protection against SARS‐CoV‐2. A cutoff of 50% vaccine efficacy (VE) has been widely used in earlier correlational analyses and modeling studies, including reports from Public Health England (PHE) and investigations examining binding antibody units (BAU/mL) in relation to breakthrough infection risk. Similarly, the 60% VE threshold has been referenced as an operational indicator of enhanced protective immunity in several prior publications.

As there is no universally accepted BAU/mL threshold for defining protective immunity, these values should be interpreted as pragmatic, literature‐based operational thresholds rather than validated clinical cut points.

### 2.3 Statistical Analysis

Statistical analyses included *t*‐test, chi‐square test, and multivariable linear regression to assess factors associated with antibody response. Variables were selected via stepwise regression. A *p* value < 0.05 was considered significant. Analyses were performed using SAS 9.4 and GraphPad Prism 5.

Because many clinical and body‐composition variables in hemodialysis patients are interrelated, we applied a stepwise multivariable regression approach to reduce multicollinearity and avoid unstable model estimates. Variables with biological plausibility or significance in univariable analysis (*p* < 0.10) were entered into the initial model. We acknowledge that several potentially important confounders—including inflammatory markers (CRP, IL‐6), nutritional indices (serum albumin, nPCR, malnutrition–inflammation score [MIS]), and medication data (e.g., corticosteroid or immunosuppressant use)—were not available for all participants and therefore could not be included in the full multivariable adjustment.

To assess robustness, we performed sensitivity analyses by re‐running the models after excluding extreme BMI values and after removing variables with high pairwise correlations, and these analyses produced results consistent with the primary findings.

Multivariable analyses used a stepwise selection procedure because of collinearity among demographic, clinical, and biochemical variables. Candidate variables with *p* < 0.10 in univariable analyses were allowed to enter the model, and variables were retained at *p* < 0.05. Model assumptions, including linearity, homoscedasticity, and absence of multicollinearity, were verified before final interpretation.

## 3 Results

### 3.1 Baseline Characteristics in Our Study Cohort

A total of 402 participants were enrolled, including 276 hemodialysis (HD) patients (mean age 65.5, 151 males) and 126 healthy controls (mean age 45.8, 58 males). HD patients were older and had more comorbidities such as congestive heart failure, cardiovascular disease, diabetes, and hypertension (Table 1).

**TABLE 1:** Demographic data between HD patients and control.

	Hemodialysis patients	Controls	*p*
	(*n* = 276)	(*n* = 126)	
Age, year	65.5 (58.2, 73.3)	45.8 (40.1, 52.4)	**< 0.001**
Male	151 (54.7%)	58 (46.0%)	0.132
Body mass index, kg/m^2^	23.6 (21.7, 26.7)	23.4 (21.8, 26.3)	0.837
Comorbidity			
Congestive heart failure	80 (29.0%)	0 (0.0%)	**< 0.001**
Acute myocardial infarction	27 (9.8%)	0 (0.0%)	**< 0.001**
Left ventricular hypertrophy	217 (78.6%)	0 (0.0%)	**< 0.001**
Cardiovascular disease	28 (10.1%)	0 (0.0%)	**< 0.001**
Cancer	63 (22.8%)	3 (2.4%)	**< 0.001**
Hypertension	259 (93.8%)	6 (4.8%)	**< 0.001**
Coronary artery disease	42 (15.2%)	0 (0.0%)	**< 0.001**
Diabetes mellitus	137 (49.6%)	3 (2.4%)	**< 0.001**
Chronic obstructive pulmonary	8 (2.9%)	0 (0.0%)	0.061
Hyperlipidemia	114 (41.3%)	6 (4.8%)	**< 0.001**

*Note:* The Mann–Whitney *U* test tested the comparison of continuous variables; the Chi‐square test tested the Comparison of category variables. Bold values indicates statistical significance *p* < 0.05.

### 3.2 Comparison Antibody Titer Response Between HD Patients and Control

**FIGURE 1:**
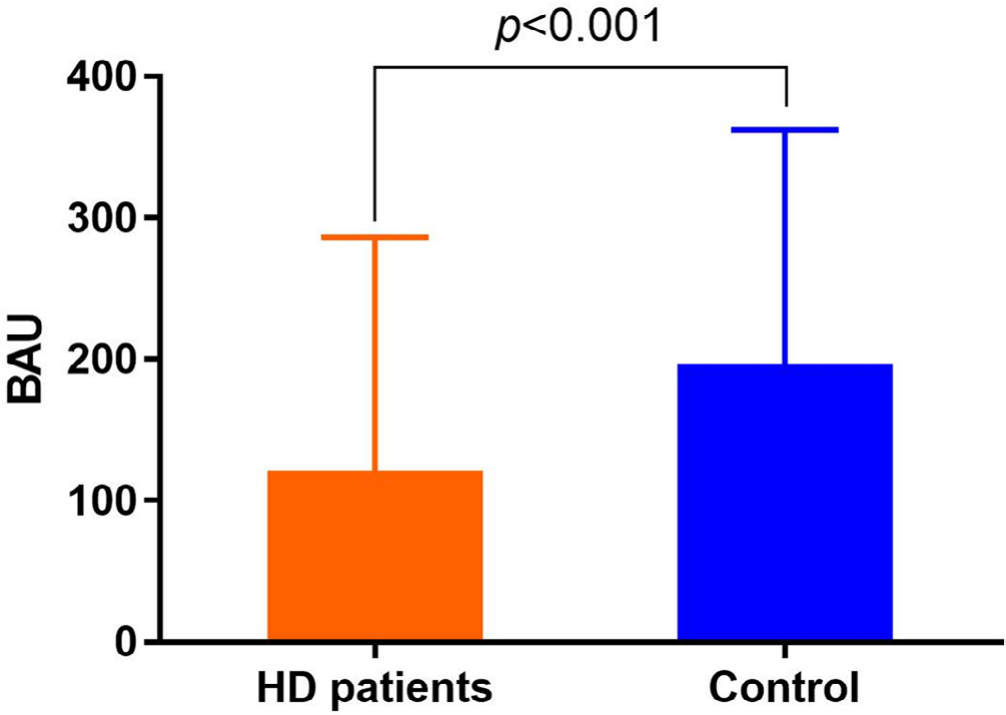
The comparison of the median of COVID‐19 antibody at 2 weeks after the second dose of Vaxzevria.

The median COVID‐19 antibody levels (BAU) after the second Vaxzevria dose were significantly lower in hemodialysis patients compared to controls (Figure 1). The mean BAU values after the second dose of vaccination were significantly lower in the hemodialysis patients (121.6 BAU/mL) compared to controls (196.9 BAU/mL), with a *p* value less than 0.001 (Table S1).

To address the substantial age difference between the HD cohort and control group, we performed age‐adjusted analyses using multivariable regression. The associations between HD status and reduced antibody response remained statistically significant after adjustment for age. In addition, age‐stratified analyses (≤ 60 vs. > 60 years) yielded similar trends, indicating that the observed group differences were not solely attributable to age imbalance.

### 3.3 Local and Specific Systemic Adverse Effects of COVID‐19 Vaccine in Our HD Study Cohort After the First and Second Doses

After the first dose of COVID‐19 vaccine, hemodialysis patients showed significantly lower rates of local pain, swelling, and bruising (29.3%), fatigue or weakness (34.1%), fever or chills (26.1%), headache (10.9%), dizziness (4.3%), abdominal pain (1.1%), arthralgia (5.1%), muscle pain (15.6%), and difficulty breathing or respiratory distress (0.7%) compared with the controls. Similarly, compared with the healthy subjects, hemodialysis patients still showed significantly less local pain, swelling, bruising, fatigue or weakness, headache, dizziness, abdominal pain, muscle pain, difficulty breathing or respiratory distress, skin itching, rash or urticaria, and no arthralgia after the second dose of COVID‐19 vaccine. (Table 2).

**TABLE 2:** Self‐reported reactions to vaccine after the first and second dose.

	First dose			Second dose		
	HD patients (*n* = 276)	Controls (*n* = 126)	*p*	HD patients (*n* = 276)	Controls (*n* = 126)	*p*
Local pain, swelling, bruising	81 (29.3%)	74 (58.7%)	**< 0.001**	32 (11.6%)	54 (42.9%)	**< 0.001**
Fatigue, weakness	94 (34.1%)	93 (73.8%)	**< 0.001**	31 (11.2%)	48 (38.1%)	**< 0.001**
Fever or chills	72 (26.1%)	77 (61.1%)	**< 0.001**	10 (3.6%)	8 (6.3%)	0.334
Headache	30 (10.9%)	54 (42.9%)	**< 0.001**	5 (1.8%)	19 (15.1%)	**< 0.001**
Dizziness	12 (4.3%)	44 (34.9%)	**< 0.001**	9 (3.3%)	18 (14.3%)	**< 0.001**
Nausea or vomiting	17 (6.2%)	14 (11.1%)	0.127	2 (0.7%)	4 (3.2%)	0.080
Abdominal pain	3 (1.1%)	9 (7.1%)	**0.002**	1 (0.4%)	2 (1.6%)	0.233
Arthralgia	14 (5.1%)	39 (31.0%)	**< 0.001**	0 (0.0%)	8 (6.3%)	**< 0.001**
Muscle pain	43 (15.6%)	72 (57.1%)	**< 0.001**	4 (1.4%)	33 (26.2%)	**< 0.001**
Difficulty breathing or respiratory distress	2 (0.7%)	6 (4.8%)	**0.013**	1 (0.4%)	5 (4.0%)	**0.013**
Skin itching, rash or urticaria	8 (2.9%)	9 (7.1%)	0.090	1 (0.4%)	4 (3.2%)	**0.035**

*Note:* The Chi‐square test tested the comparison of category variables. Bold values indicates statistical significance *p* < 0.05.

### 3.4 Contributing Factors of COVID‐19 Vaccine Responses for Hemodialysis Patients After Second Dose Vaccination

In simple linear regression, several clinical factors were positively associated with immune response (BAU) after the second COVID‐19 vaccine dose in hemodialysis patients, including BMI (*β* = 22.62, 95% CI: 12.02–33.22, *p* < 0.001), diastolic blood pressure (*β* = 3.93, 95% CI: 0.61–7.25, *p* = 0.020), white blood cell count (*β* = 25.47, 95% CI: 4.04–46.90, *p* = 0.020), cholesterol (*β* = 1.08, 95% CI: 0.08–2.09, *p* = 0.035), and creatinine (*β* = 16.66, 95% CI: 0.49–32.83, *p* = 0.044). Older age was negatively associated (*β* = −3.25, 95% CI: −6.25 to −0.25, *p* = 0.034). In multiple linear regression, BMI (*β* = 20.63, 95% CI: 9.15–32.11, *p* < 0.001), diastolic blood pressure (*β* = 3.74, 95% CI: 0.19–7.28, *p* = 0.039), and cholesterol (*β* = 1.37, 95% CI: 0.15–2.60, *p* = 0.028) remained positively associated (Table 3). Sensitivity analysis confirmed BMI (*β* = 23.59, 95% CI: 12.97–34.20, *p* < 0.001), diastolic blood pressure (*β* = 3.64, 95% CI: 0.48–6.79, *p* = 0.024), and diabetes mellitus (*β* = −105.33, 95% CI: −190.60 to −20.06, *p* = 0.016) as significant factors (Table S3).

**TABLE 3:** The relationship between clinical factors of hemodialysis patients and response to second dose vaccination in simple and multiple linear regression analysis.

	Simple	*p*	Multiple	*p*
	*β* (95% CI)		*β* (95% CI)	
Age	−3.25 (−6.25, −0.25)	**0.034**	1.03 (−3.16, 5.21)	0.629
Male	−28.79 (−103.06, 45.48)	0.446		
Body mass index	22.62 (12.02, 33.22)	**< 0.001**	20.63 (9.15, 32.11)	**< 0.001**
Systolic blood pressure	1.29 (−0.43, 3.02)	0.140		
Diastolic blood pressure	3.93 (0.61, 7.25)	**0.020**	3.74 (0.19, 7.28)	**0.039**
Hemodialysis vintage	−2.07 (−7.30, 3.15)	0.436		
Comorbidity				
Diabetes mellitus	−69.46 (−143.03, 4.10)	0.064		
Hypertension	110.22 (−43.16, 263.61)	0.158		
Hyperlipidemia	−42.69 (−117.69, 32.30)	0.263		
Coronary artery disease	−72.98 (−175.66, 29.69)	0.163		
Heart failure	−36.69 (−118.15, 44.77)	0.376		
Liver cirrhosis	−56.61 (−231.18, 117.95)	0.524		
Cancer	−32.32 (−120.42, 55.77)	0.471		
Parathyroidectomy	18.44 (−71.81, 108.69)	0.688		
Laboratory data				
White blood cells	25.47 (4.04, 46.90)	**0.020**	11.12 (−15.48, 37.73)	0.411
Hemoglobin	−11.02 (−44.58, 22.54)	0.519		
MCV	−2.14 (−6.82, 2.54)	0.368		
Platelet	0.65 (−0.02, 1.32)	0.055		
Total protein	−6.46 (−80.15, 67.23)	0.863		
Albumin	−67.50 (−173.42, 38.43)	0.211		
Aspartate aminotransferase	−1.87 (−4.59, 0.85)	0.177		
Alanine transaminase	−1.17 (−5.49, 3.15)	0.595		
Alkaline Phosphatase	−0.30 (−0.97, 0.38)	0.391		
Cholesterol	1.08 (0.08, 2.09)	**0.035**	1.37 (0.15, 2.60)	**0.028**
Triglyceride	0.22 (−0.14, 0.59)	0.223		
Blood urea nitrogen	−1.22 (−3.55, 1.11)	0.302		
Creatinine	16.66 (0.49, 32.83)	**0.044**	5.54 (−15.67, 26.75)	0.607
Uric acid	16.79 (−7.31, 40.89)	0.171		
Sodium	5.12 (−7.41, 17.65)	0.422		
Potassium	16.80 (−48.18, 81.78)	0.611		
Total Ca	3.00 (−36.14, 42.14)	0.880		

*Note:* Bold values indicates statistical significance *p* < 0.05.

**FIGURE 2:**
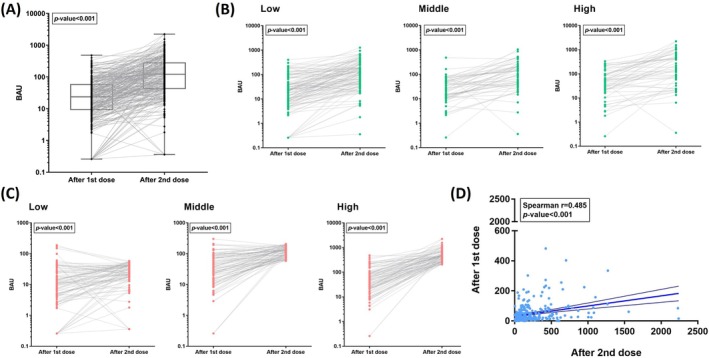
Antibody response after second dose Vaxzevria in hemodialysis patients (A) Overall hemodialysis patients (B) hemodialysis patients stratified by baseline body mass index group [Low: BMI < 24; Middle: 24 ≤ BMI < 27; High: BMI ≥ 27] (C) hemodialysis patients stratified by tertile group of antibody response after the second vaccine dose [Low: BAU ≤ 58.18; Middle: 58.18207.17] (D) The correlation between the BAU measured after the first and second dose vaccine.

Given BMI's role, the immune response was stratified by BMI. The second vaccine dose elicited a stronger response than the first (Figure 2A), with lower BMI patients (< 24 kg/m^2^) showing weaker responses after the first dose (Figure 2B). Poor responders were also observed in the lowest tertile of BAU levels (Figure 2C), while higher BMI was associated with a stronger immune response in post hoc ANOVA (Table S2). A moderate correlation was found between BAU levels of the first and second doses (*r* = 0.485, *p* < 0.001, Figure 2D). Different weight measures in hemodialysis patients (dry, pre‐, and post‐dialysis) showed persistent positive associations with BAU after the second dose in fully adjusted models (*β* = 8.56–8.62, *p* < 0.001). Notably, weight decrease during hemodialysis was also positively associated (Table 4). Overall, the second vaccine dose elicited a stronger immune response in hemodialysis patients, but those with low BMI had poorer responses. Higher antibody responders (BAU > 207.17) were significantly younger (61.7 ± 12.4 years, *p* = 0.002) and had higher systolic and diastolic blood pressure (Table S2).

**TABLE 4:** The relationship between body weight and ultrafiltration amount of hemodialysis patients and response to second dose vaccination in linear regression model.

	Crude	*p*	Adjusted model 1	*p*	Adjusted model 2	*p*
	*β* (95% CI)		*β* (95% CI)		*β* (95% CI)	
Dry weight, kg	4.09 (1.67, 6.52)	**0.001**	4.94 (2.23, 7.65)	**< 0.001**	8.56 (4.69, 12.43)	**< 0.001**
Pre‐dialysis weight, kg	4.05 (1.71, 6.38)	**< 0.001**	4.82 (2.22, 7.43)	**< 0.001**	8.41 (4.68, 12.13)	**< 0.001**
Post‐dialysis weight, kg	4.10 (1.69, 6.51)	**< 0.001**	4.91 (2.22, 7.59)	**< 0.001**	8.62 (4.76, 12.47)	**< 0.001**
Body weight decrease, kg	59.48 (18.87, 100.08)	**0.004**	55.71 (12.10, 99.33)	**0.012**	73.99 (18.42, 129.55)	**0.009**

*Note:* Adjusted model 1: adjusted age and gender. Adjusted model 2: adjusted age and gender, hemodialysis vintage, systolic BP, diastolic BP, and comorbidities. Bold values indicates statistical significance *p* < 0.05.

While BMI was associated with immune response (BAU) after the second COVID‐19 vaccine dose in hemodialysis patients, its limitations include inadequate body composition assessment and inability to distinguish between fat and muscle. Further analysis showed that BAU was positively associated with FTI (*β* = 18.55, 95% CI: 8.56–28.55, *p* < 0.001), TBW (*β* = 9.63, 95% CI: 1.01–18.24, *p* = 0.029), and extracellular water (*β* = 21.16, 95% CI: 3.54–38.79, *p* = 0.019) (Table 5). Additionally, the ECW/ICW ratio was significantly lower in the high BAU group than in the low BAU group (*p* = 0.044, trend *p* = 0.034) (Table S4).

**TABLE 5:** The relationship between body composition monitor (BCM) parameters of hemodialysis patients and response to second dose vaccination in linear regression model.

	Crude	*p*	Adjusted model 1	*p*	Adjusted model 2	*p*
	*β* (95% CI)		*β* (95% CI)		*β* (95% CI)	
Lean tissue index	6.72 (−5.30, 18.75)	0.272	9.59 (−5.53, 24.71)	0.212	4.31 (−12.11, 20.73)	0.605
Fat tissue index	11.54 (2.73, 20.34)	**0.010**	12.64 (3.36, 21.91)	**0.008**	18.55 (8.56, 28.55)	**< 0.001**
Total body water	5.14 (−0.39, 10.67)	0.068	10.51 (2.41, 18.60)	**0.011**	9.63 (1.01, 18.24)	**0.029**
Intracellular water	8.32 (−1.07, 17.71)	0.082	16.35 (2.60, 30.10)	**0.020**	13.11 (−1.54, 27.76)	0.079
Extracellular water	10.83 (−1.33, 22.98)	0.081	19.23 (2.96, 35.49)	**0.021**	21.16 (3.54, 38.79)	**0.019**
Extracellular/intracellular ratio	37.17 (−306.31, 380.64)	0.831	97.23 (−269.59, 464.06)	0.602	280.99 (−133.52, 695.50)	0.183
Overhydration	−13.13 (−41.17, 14.91)	0.357	−15.13 (−43.92, 13.66)	0.301	−14.19 (−47.15, 18.77)	0.397
Hydration Status	−5.37 (−11.63, 0.89)	0.092	−5.60 (−11.86, 0.66)	0.079	−5.62 (−12.49, 1.26)	0.109

*Note:* Adjusted model 1: adjusted age and gender. Adjusted model 2: adjusted age and gender, hemodialysis vintage, systolic BP, diastolic BP, and comorbidities. Bold values indicates statistical significance *p* < 0.05.

### 3.5 Antibody Response Influences by Clinical Factors in Hemodialysis Patients

**FIGURE 3:**
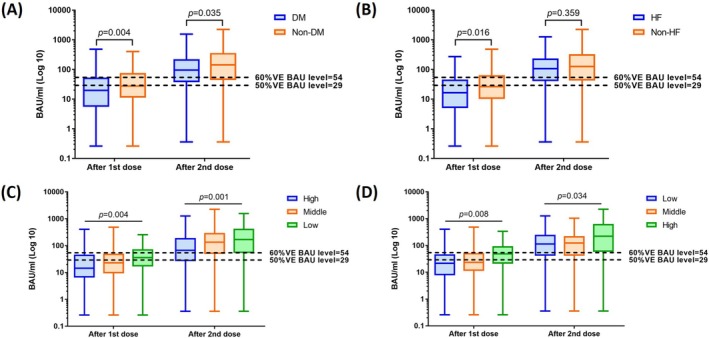
Antibody response after second dose Vaxzevria in hemodialysis patients (A) Diabetes mellitus (B) Heart failure (C) hemodialysis patients stratified by tertile group of age [Low: Age < 61.575; Middle: 61.575 ≤ Age < 70.533; High: Age ≥ 70.533] (D) hemodialysis patients stratified by baseline body mass index group [Low: BMI < 24; Middle: 24 ≤ BMI < 27; High: BMI ≥ 27].

The antibody response following the second dose of the Vaxzevria in hemodialysis patients was influenced by several clinical factors. Patients with DM exhibited significantly lower antibody levels compared to those without DM, with median BAU of 94.5 and 138.7, respectively (*p* < 0.05) (Figure 3A). Similarly, hemodialysis patients with heart failure demonstrated reduced immune responses, with a median BAU of 86.3 compared to 131.4 in patients without heart failure (*p* < 0.05) (Figure 3B). Age also played a critical role, as stratification into tertiles revealed a significant decline in antibody response with increasing age: patients in the youngest tertile had the highest median BAU (152.4), while those in the oldest tertile had the lowest median BAU (98.3, p for trend < 0.001) (Figure 3C). Additionally, baseline BMI was positively correlated with antibody response; patients in the highest BMI group (≥ 27 kg/m^2^) exhibited a median BAU of 157.2, significantly higher than the 104.8 observed in the lowest BMI group (< 24 kg/m^2^, *p* < 0.01) (Figure 3D). These findings underscore the importance of comorbidities, age, and BMI in determining vaccine efficacy in hemodialysis patients, highlighting the need for tailored immunization strategies in this vulnerable population.

In this study, two thresholds were defined to assess antibody immune responses as indicators of protection: the 50% vaccine efficacy (VE) BAU level and the 60% VE BAU level. These thresholds were used to evaluate the proportion of hemodialysis patients achieving protective immune responses after the second dose of the Vaxzevria. Among all HD patients, 46.7% (129/276) achieved antibody levels above the 50% VE threshold, while only 23.2% (64/276) surpassed the 60% VE threshold. Stratification by clinical factors revealed significant differences in protection rates. DM patients had markedly lower proportions, reaching the 50% and 60% VE thresholds compared to those without DM (35.2% vs. 55.8% and 14.8% vs. 29.1%, respectively; both *p* < 0.01) (Figure 3A). Similarly, patients with heart failure were less likely to achieve protective antibody levels, with only 31.3% and 12.5% exceeding the 50% and 60% VE thresholds, respectively, compared to 51.8% and 25.5% in those without heart failure (*p* < 0.05) (Figure 3B). Age stratification revealed that the proportion of patients achieving protective antibody levels decreased significantly with increasing age. In the youngest tertile, 57.8% and 30.2% reached the 50% and 60% VE thresholds, respectively. These proportions dropped to 43.5% and 20.7% in the middle tertile and further to 39.1% and 18.5% in the oldest tertile (*p* < 0.01) (Figure 3C). Conversely, baseline BMI showed a positive association with immune protection. In the highest BMI group (≥ 27 kg/m^2^), 53.3% and 28.6% of patients exceeded the 50% and 60% VE thresholds, respectively, compared to 41.3% and 17.4% in the lowest BMI group (< 24 kg/m^2^, *p* < 0.01) (Figure 3D). These results highlight significant variability in vaccine‐induced antibody protection among hemodialysis patients, with age, BMI, and comorbidities such as diabetes and heart failure emerging as critical factors influencing the likelihood of achieving protective immunity. These findings underscore the importance of tailored vaccination strategies to enhance immune protection in vulnerable subgroups within the hemodialysis population.

## 4 Discussion

The present study expands on previous vaccine‐immunogenicity research by providing results from a relatively large and ethnically homogeneous Asian HD cohort, an underrepresented population in prior ChAdOx1 studies. Furthermore, our analysis incorporates an integrated assessment of BMI and objective body‐composition indices (FTI, TBW, ECW/ICW) rather than evaluating these variables separately, thereby offering a more comprehensive perspective on how nutritional and fluid‐status parameters may relate to vaccine response in chronic dialysis patients. These additions address an important evidence gap and enhance the relevance of our findings for Asian and other non‐Western HD populations.

This study demonstrates significant variability in vaccine‐induced immune responses among hemodialysis patients, influenced by clinical factors such as age, BMI, and comorbidities. Hemodialysis patients exhibited significantly lower antibody levels compared to healthy controls after the second dose of the Vaxzevria. Hemodialysis patients with DM and heart failure were associated with poorer antibody responses and lower rates of achieving protective immunity, as defined by 50% and 60% VE thresholds. Age was inversely associated with antibody levels, with older patients demonstrating reduced immune responses and protection rates, while higher BMI was positively correlated with stronger antibody responses and a greater likelihood of surpassing protection thresholds. Additionally, body composition analysis revealed that FTI and TBW were positively associated with antibody responses, whereas lean tissue index and hydration status showed no significant correlation. These findings underscore the vulnerability of hemodialysis patients to suboptimal vaccine‐induced immunity, particularly in those with advanced age or comorbidities, highlighting the need for targeted vaccination strategies and potential booster doses to optimize protection in this high‐risk population. In our study, higher BMI was associated with a stronger antibody response, but this relationship should be interpreted as a correlation rather than a causal effect of adiposity. The observed association may instead reflect better nutritional reserves, lower inflammation, or overall metabolic stability, which are more common in HD patients with higher BMI. Therefore, BMI may act as a proxy indicator of nutritional or inflammatory status rather than functioning as an independent biological enhancer of vaccine immunogenicity.

Our findings align with immunogenicity reports from dialysis cohorts receiving mRNA vaccines, which similarly identified age, diabetes, and nutritional status as determinants of antibody response. Studies evaluating heterologous or mixed vaccination regimens have also demonstrated enhanced immunogenicity; however, the present study focused on homologous ChAdOx1 vaccination, highlighting the need for future comparative evaluations across different vaccine platforms.

A previous study in French showed that patients with ESRD have been associated with an increased risk of severe disease, hospitalization, and mortality attributed to COVID‐19 infection compared to the general population [21]. In addition, previous studies also indicated a potentially diminished antibody response in ESKD patients compared to healthy individuals [9, 10]. Immune response to the COVID‐19 vaccine is significantly lower in the hemodialysis patient group compared to the control group, as shown in our study. We found that hemodialysis patients had significantly lower median COVID‐19 antibody levels after the second dose of the Vaxzevria compared to controls (121.6 BAU/mL vs. 196.9 BAU/mL, *p* < 0.001). Thus, hemodialysis patients may require closer monitoring or additional vaccine doses to achieve a comparable level of antibody protection against COVID‐19 as the general population. As for adverse events of COVID‐19 vaccination reported previously, injection site reactions, fatigue, fever, myalgia, arthralgia, and headache are commonly observed in non‐dialysis CKD patients. Another study of COVID‐19 vaccination on hemodialysis patients also showed that the adverse events experienced were predominantly minor, short‐lived, and affected less than 5% of individuals' daily activities [22]. In the present study, hemodialysis patients presented significantly lower rates of side effects after both the first and second doses of the COVID‐19 vaccine compared to control participants, which is similar to the previous report.

Previous studies have also concluded that age was an important factor in the humoral response following the vaccine [8, 23, 24, 25]. Our findings of a lower immune response in the oldest group of vaccinated hemodialysis patients compared to other age groups in our study were similar to findings in previous literature; there was a significant inverse correlation between older age and antibody levels in both study groups. Across all age groups, the control group had consistently higher antibody levels than the dialysis group, with this difference being statistically significant in people aged 60–70 years [8]. As for comorbidities, recent research has indicated an impaired response to vaccination in individuals with type 2 diabetes primarily due to defects in T‐cell function and related impairment of B‐cell activation [26, 27]. People with diabetes have compromised innate and adaptive immune systems. T cells, in particular, have been reported to be abnormally differentiated in those with T2DM. Patients with hyperglycemia exhibit a reduced number of circulating helper T cells, increased numbers of senescent T cells, impaired T‐cell migration, and decreased T‐cell cytolytic activity. These T‐cell abnormalities in diabetes reduce protective T‐ and B‐cell responses against viral pathogens, including SARS‐CoV‐2, during infection [16]. Thus, it is reasonable to find hemodialysis patients with diabetes mellitus had significantly lower median antibody levels compared to those without diabetes after the first dose and second dose in our study. As for hemodialysis patients with heart failure, a reduced immune response was found compared to those without heart failure. This novel finding was not reported in previous COVID‐19 vaccine studies. However, it was reported that patients with heart failure have lower initial antibody responses to the influenza vaccine compared to healthy individuals [17], which may be partially analogous to our hemodialysis population. Activation of the sympathetic nervous system has detrimental effects on the myocardium but may also contribute to a diminished response to the influenza vaccine in patients with heart failure [18]. Further study is still needed to confirm our findings.

The mortality rate among dialysis patients is notably elevated when they contract COVID‐19 [19], this may be related to the limited effectiveness of vaccination in such groups due to weakened immune responses. With respect to antibody response in hemodialysis patients, a study from Portugal showed that patients with a higher BMI showed a better immune response in the hemolysis group but an insignificant association between BMI and antibody response [28]. Several studies suggest that obesity is associated with lower antibody titers after COVID‐19 vaccination compared to individuals with a normal BMI in the general population [29, 30, 31], but similar specific studies in hemodialysis patients are lacking. Contrary to the general population, we demonstrate a better COVID‐19 vaccination antibody immune response in hemodialysis with higher BMI. It is a puzzling phenomenon, but it should be explainable. Obesity is linked to better survival in hemodialysis patients, which is called the “obesity‐survival paradox” [32], it was observed that heavier patients were considered obese (BMI ≥ 27 kg/m^2^). Since we observed a better immune response in the first and second doses of COVID‐19 vaccination in hemodialysis patients, our finding could be explained by a better nutrient status among the higher BMI group.

The BMI paradox in hemodialysis patients presents an intriguing connection to the COVID‐19 vaccine immune response. While higher BMI typically indicates poorer health outcomes in the general population, hemodialysis patients with elevated BMI often demonstrate better survival rates, creating a paradoxical relationship [33]. This phenomenon is particularly relevant when examining vaccine effectiveness through complex immunological pathways. Low BMI in hemodialysis patients often indicates malnutrition‐inflammation complex syndrome (MICS), which suppresses immune function and weakens antibody responses to vaccines [34]. Furthermore, studies have shown that hemodialysis patients generally have a weaker immune response to vaccines compared to healthy controls, which could be influenced by factors like nutritional status and BMI [11, 35]. The uremic state in hemodialysis patients already compromises immune function, typically resulting in reduced vaccine responses, and this is further exacerbated by protein‐energy wasting (PEW) in lower BMI patients, leading to compromised vaccine responses. In contrast, a higher BMI is associated with better nutritional status and a more favorable cytokine profile. The increased adipose tissue in patients with higher BMI functions as an endocrine organ, producing adipokines and inflammatory mediators that modify the immune response. This altered inflammatory state, while potentially detrimental in some contexts, may actually influence the antibody response to COVID‐19 vaccination in unique ways [36]. Clinically, this paradox highlights the importance of considering BMI when evaluating vaccine efficacy in hemodialysis patients. Lower BMI patients may require additional vaccine doses or adjuvants to enhance immune responses while addressing malnutrition and inflammation could improve outcomes across all BMI categories. The complex interplay between nutritional status, inflammatory factors, and the uremic environment warrants further research to optimize vaccination strategies for this vulnerable population.

Since we observe a BMI paradox and COVID‐19 vaccine antibody response in hemodialysis patients, further body composition parameters are helpful to dissect the complex relationship. In our study, FTI, TBW, extracellular water, and the extracellular to intracellular water (ECW/ICW) ratio demonstrated significant associations with vaccine immune response through various physiological pathways. Fat tissue plays a particularly crucial role in this relationship. Adipose tissue acts as a dynamic endocrine organ, secreting various molecules known as adipokines and cytokines. These secretions are involved in multiple physiological processes, including inflammation, metabolism, and immune regulation [37]. The role of TBW and extracellular water is particularly complex due to hemodialysis patients' altered fluid homeostasis. While these patients undergo regular fluid removal through dialysis, maintaining appropriate TBW supports lymphatic system function and immune cell trafficking between dialysis sessions. Extracellular water provides the essential medium for immune cell migration, cytokine and antibody transport, and effective antigen presentation to immune cells [38]. However, persistently elevated extracellular water levels might signify ongoing chronic inflammation, which could counteract these benefits. The ECW/ICW ratio holds special significance as it reflects not only fluid status but also indirectly reflects nutritional and inflammatory status, crucial factors for immune competence. Hemodialysis patients often experience MICS, which can significantly impact immune function [39]. Better balanced ECW/ICW ratios often correlate with improved protein‐energy reserves and reduced MICS, supporting the production of antibodies and proliferation of immune cells after vaccination [40]. Clinically, these findings underscore the importance of monitoring and optimizing body composition parameters in hemodialysis patients to help identify patients at higher risk of poor vaccine responses, enabling personalized strategies such as additional vaccine doses or the use of adjuvants. Further studies are warranted to explore the mechanisms and clinical interventions that could optimize vaccine outcomes in this high‐risk group.

Associations between higher BMI or favorable body‐composition profiles and stronger antibody responses may reflect immune–nutritional interactions. Prior studies have suggested that adipokines, metabolic reserves, and inflammatory regulation influence vaccine immunogenicity, although our study did not directly measure these pathways. Furthermore, ECW/ICW imbalance has been linked to chronic inflammation in hemodialysis patients and may partially explain the correlations observed.

Several potential mechanisms may explain the associations we observed; however, these interpretations should be considered hypothesis‐generating rather than conclusive. Prior studies have suggested that adipokines, nutritional reserves, and inflammatory regulation could influence vaccine responsiveness, which may partially relate to the positive association between BMI and antibody levels seen in our cohort. Likewise, alterations in extracellular and intracellular water distribution have been linked to systemic inflammation in hemodialysis patients, providing a possible explanation for the associations involving ECW/ICW.

Importantly, our study did not measure adipokines, inflammatory cytokines, hydration‐related biomarkers, or other mechanistic parameters, and therefore we cannot directly confirm these pathways. The discussion of adipokines, extracellular water balance, and the “obesity‐survival paradox” reflects hypotheses derived from prior literature, rather than mechanisms demonstrated in our dataset.

Given these limitations, the mechanistic interpretations presented here should be viewed as speculative and requiring validation. Future prospective studies incorporating mechanistic biomarkers and longitudinal assessments are needed to verify these proposed pathways.

In conclusion, this study reveals that vaccine‐induced immunity in hemodialysis patients is influenced by age, BMI, body composition, and comorbidities, with older age and conditions like diabetes associated with reduced responses. Tailored vaccination strategies and booster doses are essential to improve protection in this vulnerable population.

## 5 Limitation

Our study has several limitations. First, residual confounding cannot be excluded, as we were unable to obtain complete data on key inflammatory and nutritional markers such as CRP, IL‐6, serum albumin, nPCR, and the malnutrition–inflammation score (MIS). In addition, information on corticosteroid or other immunosuppressive medication use was incomplete and could not be incorporated into the multivariable models. The absence of these variables may have resulted in partial adjustment of the regression analyses. Although we attempted to mitigate this issue through stepwise modeling and sensitivity analyses, our results should be interpreted with caution, and future studies incorporating comprehensive biochemical and medication data are needed to confirm these findings. Another important limitation is the age imbalance between the hemodialysis patients and healthy controls. Because age is a well‐established determinant of vaccine immunogenicity, the younger control group could partially exaggerate the apparent difference in antibody response. Although we adjusted for age in all multivariable models and confirmed similar findings in age‐stratified analyses, residual confounding due to age‐related immune variation cannot be completely excluded. Future studies with age‐matched control groups or longitudinal within‐patient designs would provide more definitive conclusions.

In addition, body‐composition parameters were available only for a convenience subsample of the hemodialysis cohort, rather than for all participants. Because BCM assessment was limited by equipment availability and scheduling constraints, the resulting subsample may not be fully representative of the entire HD population. This may introduce selection bias, particularly if nutritional, hydration, or inflammatory profiles differ between patients who did and did not undergo BCM measurement.

Therefore, the findings involving FTI, TBW, or ECW/ICW should be interpreted with caution and considered exploratory.

Another limitation concerns the use of 50% and 60% VE antibody thresholds. Although these cutoffs have been referenced in prior studies and public‐health reports as practical correlates of protection, they are not universally validated and may not apply uniformly across populations, particularly in immunocompromised patients such as those receiving hemodialysis. The thresholds used in this study therefore represent operational definitions based on previous literature rather than established clinical standards.

Consequently, the interpretation of “protective” antibody levels should be approached with caution, and future cohort studies linking BAU/mL directly to clinical breakthrough infections in HD populations are needed to refine these thresholds.

Data on inflammatory biomarkers (e.g., CRP, IL‐6), dialysis adequacy indices, and comprehensive nutritional indicators (e.g., MIS, normalized protein catabolic rate) were not available for all patients, limiting adjustment for these potential confounders. The possibility of residual confounding by unmeasured inflammatory or dialysis‐related variables cannot be excluded.

## Funding

The study was funded by grants from the Ministry of Science and Technology, Taiwan (MOST 111‐2314‐B‐037‐032‐MY3), Kaohsiung Medical University Hospital, Taiwan (KMUH111‐1M60, KMUH111‐1R73, KMUH111‐1M09, KMUH110‐0M13, KMUH110‐0M73, KMUH110‐0M12, and KMUH‐DK(C)112001), and Kaohsiung Medical University, Taiwan (KT113P006, KT112P012, NYCUKMU‐112‐I006, NHRIKMU‐111‐I003, and NHRIKMU‐111‐I001). This study is supported partially by the Kaohsiung Medical University Research Center Grant (KMU‐TC112B04), KMUH‐DK(C)112001, and KMUH‐DK(C)113003.

## Ethics Statement

This study was approved by the Institutional Review Board of Kaohsiung Medical University (KMUHIRB‐E(II)‐20210161). Informed consent was obtained from all participants.

## Conflicts of Interest

The authors declare no conflicts of interest.

## Supporting information


**Table S1:** The comparison of COVID‐19 antibody 2 weeks after the second dose of AZ vaccination.
**Table S2:** Characteristics of the hemodialysis participants according to the antibody response after the second vaccine dose.
**Table S3:** The relationship between clinical factors of hemodialysis patients and response to second dose vaccination in multiple linear regression analysis with stepwise procedure.
**Table S4:** Body composition monitor information of the hemodialysis participants according to the antibody response after the second vaccine dose.

## Data Availability

The anonymized data supporting the findings of this study, including demographic information, clinical characteristics, body composition parameters, and antibody response measurements from 276 hemodialysis patients and 126 controls following Vaxzevria vaccination, are available from the corresponding author upon reasonable request. Access to individual patient data may be restricted to protect participant privacy. The statistical code used for linear regression modeling and stratification analyses is also available upon request. Any data sharing will be subject to institutional review board approval and will require a formal data sharing agreement.
