# Supplementary material for: Identification of Clinical Factors Associated With the Immunogenicity of Homologous ChAdOx1‐nCoV‐19 Vaccine in Hemodialysis Patients
Source: Kaohsiung J Med Sci. 2026 Jan 19:e70173. Online ahead of print. doi: 10.1002/kjm2.70173 (PMC13399820; doi:10.1002/kjm2.70173)
Supplement: Supplementary file 1 — Table S1: The comparison of COVID‐19 antibody 2 weeks after the second dose of AZ vaccination. Table S2: Characteristics of the hemodialysis participants according to the antibody response after the second vaccine dose. Table S3: The relationship between clinical factors of hemodialysis patients and response to second dose vaccination in multiple linear regression analysis with stepwise procedure. Table S4: Body composition monitor information of the hemodialysis participants according to the antibody response after the second vaccine dose. [file KJM2-9999-e70173-s001.pdf]

Supplementary Table 1. The comparison of COVID-19 antibody 2 weeks after the second dose of AZ vaccination

	Hemodialysis patients (n=276)	Controls (n=126)	*p*-value
BAU after the second dose of vaccination	121.6 (41.8, 285.3)	196.9 (118.1, 362.2)	**<0.001**

The Mann-Whitney U test tested the comparison of continuous variables.

Supplementary Table 2. Characteristics of the hemodialysis participants according to the antibody response after the second vaccine dose

	All (n=276)	Low (BAU≤58.18) (n=92)	Middle (58.18<BAU≤207.17) (n=92)	High (BAU>207.17) (n=92)	*p*-value	*p* for trend
Age, year	64.5 ± 12.3	68.0 ± 11.2	63.9 ± 12.4	61.7 ± 12.4*	**0.002**	**<0.001**
Male	151 (54.7%)	49 (53.3%)	54 (58.7%)	48 (52.2%)	0.636	0.882
Body mass index, kg/m^2	24.4 ± 4.0	24.2 ± 3.4	23.7 ± 4.4	25.4 ± 4.0†	**0.040**	0.072
Systolic BP, mmHg	146.3 ± 25.6	139.9 ± 23.9	146.5 ± 27.3	152.5 ± 24.2*	**0.011**	**0.003**
Diastolic BP, mmHg	80.1 ± 13.2	76.5 ± 11.7	80.3 ± 14.1	83.4 ± 13.0*	**0.006**	**0.001**
Hemodialysis vintage, years	5.9 (2.5, 11.1)	5.8 (2.5, 10.0)	6.8 (2.8, 13.3)	5.4 (2.3, 12.2)	0.431	0.843
Comorbidities						
Diabetes mellitus	137 (49.6%)	50 (54.3%)	49 (53.3%)	38 (41.3%)	0.145	0.077
Hypertension	259 (93.8%)	87 (94.6%)	82 (89.1%)	90 (97.8%)	**0.046**	0.358
Hyperlipidemia	114 (41.3%)	39 (42.4%)	45 (48.9%)	30 (32.6%)	0.078	0.178
Coronary artery disease	42 (15.2%)	19 (20.7%)	12 (13.0%)	11 (12.0%)	0.202	0.101
Heart failure	80 (29.0%)	28 (30.4%)	27 (29.3%)	25 (27.2%)	0.884	0.626
Liver cirrhosis	13 (4.7%)	4 (4.3%)	6 (6.5%)	3 (3.3%)	0.683	0.728
Cancer	63 (22.8%)	26 (28.3%)	22 (23.9%)	15 (16.3%)	0.148	0.053
Parathyroidectomy	59 (21.4%)	14 (15.2%)	26 (28.3%)	19 (20.7%)	0.095	0.369
Laboratory data						
White blood cells, 10^3/uL	5.7 (4.8, 6.8)	5.7 (4.7, 6.8)	5.6 (4.4, 6.7)	6.0 (5.1, 7.0)	0.190	0.198
Hemoglobin, mg/dl	10.9 (10.0, 11.4)	10.8 (9.9, 11.4)	11.1 (10.4, 11.6)	10.9 (10.1, 11.5)	0.109	0.365

MCV, fl	91.3 (86.7, 95.2)	92.0 (87.4, 95.6)	90.1 (86.5, 94.1)	91.4 (86.9, 95.3)	0.384	0.575
Platelet, 10^3/uL	170.0 (138.0, 207.5)	167.5 (138.0, 205.0)	167.0 (134.0, 211.5)	178.5 (142.0, 208.0)	0.562	0.352
Total protein, g/dL	6.7 ± 0.5	6.6 ± 0.5	6.8 ± 0.5	6.7 ± 0.5	0.057	0.198
Albumin, g/dL	3.8 ± 0.3	3.8 ± 0.4	3.8 ± 0.3	3.8 ± 0.4	0.675	0.789
AST, IU/L	16.7 (13.5, 20.4)	16.2 (12.8, 20.5)	17.6 (14.3, 21.0)	16.7 (13.4, 19.3)	0.280	0.764
ALT, IU/L	12.4 (10.0, 17.0)	11.6 (9.6, 15.9)	12.9 (10.5, 17.2)	12.8 (9.9, 17.3)	0.290	0.354
Alkaline Phosphatase, IU/L	75.9 (61.4, 95.4)	75.9 (63.6, 97.0)	78.7 (59.1, 96.8)	75.1 (60.9, 89.7)	0.603	0.311
Cholesterol, mg/dL	153.7 (129.7, 174.5)	151.1 (132.2, 169.9)	146.5 (128.7, 170.5)	159.8 (130.1, 182.2)	0.266	0.290
Triglyceride, mg/dL	116.9 (78.1, 181.7)	118.2 (74.1, 186.2)	104.7 (72.5, 164.1)	128.5 (89.8, 187.1)	0.093	0.159
BUN-B, mg/dL	67.2 ± 15.9	66.9 ± 16.0	68.6 ± 16.3	66.0 ± 15.4	0.523	0.690
Creatinine, mg/dL	9.7 ± 2.3	9.3 ± 2.2	9.8 ± 2.1	10.1 ± 2.5	0.058	**0.017**
Uric acid, mg/dl	6.4 (5.5, 7.4)	6.3 (5.4, 7.2)	6.4 (5.5, 7.2)	6.6 (5.8, 7.6)	0.188	0.078
Sodium, mmol/L	135.8 (134.2, 137.8)	135.7 (134.0, 137.7)	135.8 (133.6, 137.7)	135.9 (134.6, 137.9)	0.702	0.592
Potassium, mmol/L	4.2 ± 0.6	4.2 ± 0.6	4.3 ± 0.6	4.3 ± 0.5	0.675	0.401
Total Ca, mg/dl	9.1 ± 0.9	9.2 ± 1.0	9.0 ± 1.0	9.0 ± 0.9	0.352	0.249
Phosphorus, mg/dl	4.8 ± 1.3	4.7 ± 1.2	4.7 ± 1.3	4.9 ± 1.3	0.654	0.454
Total Ca*P	43.2 ± 11.5	43.4 ± 11.4	42.5 ± 12.4	43.7 ± 10.9	0.741	0.832
Urea reduction ratio	0.7 (0.7, 0.8)	0.7 (0.7, 0.8)	0.7 (0.7, 0.8)	0.7 (0.7, 0.8)	0.494	0.834
Kt/V (D)	1.6 ± 0.2	1.6 ± 0.2	1.7 ± 0.2	1.6 ± 0.3	0.345	0.652
nPCR	1.1 (0.9, 1.2)	1.0 (0.9, 1.2)	1.1 (0.9, 1.2)	1.0 (0.9, 1.2)	0.224	0.877
Iron, μg/dl	60.0 (47.5, 75.0)	59.5 (49.5, 74.5)	61.5 (49.0, 75.0)	58.0 (46.0, 74.0)	0.582	0.427
UIBC, mg/dl	142.5 (120.1, 169.5)	142.0 (119.4, 166.0)	139.2 (115.9, 168.7)	147.5 (123.8, 172.6)	0.460	0.311

Ferritin, ng/mL	266.6 (114.7, 415.6)	247.4 (148.5, 417.8)	281.4 (104.8, 410.4)	263.6 (103.0, 428.4)	0.859	0.981
Aluminum, μg/L	24.9 (18.2, 33.8)	25.2 (18.3, 32.8)	22.4 (17.2, 33.6)	27.9 (20.5, 34.0)	0.123	0.380
Magnesium, mg/dl	2.5 ± 0.3	2.5 ± 0.4	2.5 ± 0.3	2.5 ± 0.3	0.877	0.832
Parathyroid hormone, pg/ml	269.9 (124.6, 497.0)	246.0 (125.9, 485.9)	316.3 (166.8, 480.1)	237.8 (103.6, 500.8)	0.615	0.660
BAU/ml						
After the first dose of vaccination	23.6 (9.2, 59.5)	10.1 (3.8, 24.1)	30.5 (13.0, 74.8)	40.7 (20.4, 86.3)	**<0.001**	**<0.001**
After the second dose of vaccination	121.6 (41.8, 285.3)	26.6 (17.1, 41.8)	121.6 (83.5, 157.7)	425.6 (285.3, 630.5)	**<0.001**	**<0.001**

One-way ANOVA or Kruskal Wallis Test tested the comparison of continuous variables between antibody tertile groups.

The Chi-square test tested the comparison of category variables between antibody tertile groups.

Abbreviation: Binding antibody units, BAU; Mean corpuscular volume, MCV; Aspartate aminotransferase, AST; Alanine transaminase, ALT; Blood urea nitrogen, BUN; normalized protein catabolic rate, nPCR; Unsaturated iron-binding capacity, UIBC

Supplement Table 3. The relationship between clinical factors of hemodialysis patients and response to second dose vaccination in multiple linear regression analysis with stepwise procedure

	β (95%CI)	*p*-value
Body mass index	23.59 (12.97, 34.20)	**<0.001**
Diastolic BP	3.64 (0.48, 6.79)	**0.024**
Comorbidity		
Diabetes mellitus	-105.33 (-190.60, -20.06)	**0.016**

Adjusted for stepwise procedure selected covariates

Supplementary Table 4. Body composition monitor information of the hemodialysis participants according to the antibody response after the second vaccine dose

	All (n=276)	Low (BAU≤58.18) (n=92)	Middle (58.18<BAU≤207.17) (n=92)	High (BAU>207.17) (n=92)	p-value	p for trend
Lean tissue index, kg/m^2	13.8 (11.5, 16.8)	13.3 (10.8, 15.8)	14.0 (12.0, 16.1)	14.3 (11.5, 18.0)	0.115	**0.038**
Fat tissue index, kg/m^2	9.1 (5.8, 12.4)	9.7 (6.4, 13.1)	8.4 (5.5, 10.8)	9.1 (5.5, 13.2)	0.241	0.652
Total body water, L	33.8 (27.8, 39.0)	33.4 (27.5, 38.4)	34.0 (28.0, 37.5)	34.7 (28.5, 43.0)	0.315	0.174
Intracellular water, L	17.5 (14.3, 20.8)	16.6 (14.0, 20.2)	17.5 (14.5, 20.2)	17.9 (14.7, 23.3)	0.254	0.095
Extracellular water, L	15.9 (13.5, 18.3)	16.0 (13.6, 17.8)	15.6 (13.2, 18.0)	17.0 (14.1, 19.9)	0.217	0.225
Extracellular/Intracellular ratio, %	0.9 (0.8, 1.0)	1.0 (0.9, 1.1)	0.9 (0.8, 1.0)	0.9 (0.8, 1.0)	**0.044**	**0.034**
Overhydration, L	1.8 ± 1.6	1.8 ± 1.6	1.7 ± 1.7	1.8 ± 1.5	0.961	0.927
Hydration Status	11.2 ± 7.0	12.4 ± 7.4	10.6 ± 6.7	10.6 ± 6.9	0.207	0.123
